# The MRC National Survey of Health and Development reaches age 70: maintaining participation at older ages in a birth cohort study

**DOI:** 10.1007/s10654-016-0217-8

**Published:** 2016-12-19

**Authors:** Diana Kuh, Andrew Wong, Imran Shah, Adam Moore, Maria Popham, Philip Curran, Daniel Davis, Nikhil Sharma, Marcus Richards, Mai Stafford, Rebecca Hardy, Rachel Cooper

**Affiliations:** MRC Unit for Lifelong Health and Ageing at UCL, 33 Bedford Place, London, WC1B 5JU UK

**Keywords:** Participation, Birth cohort, Attrition, Older adults, Longitudinal study, Life course epidemiology

## Abstract

A life course approach to ageing relies on maintaining participation rates in national birth cohorts and other long-term longitudinal studies. This reduces the risk of selective attrition biasing associations between lifetime risk factors and health outcomes in later life and ensures the studies remain as representative as possible of the original population. We report the participation patterns for a postal questionnaire and home visit at 68–69 years of study members in the MRC National Survey of Health and Development, the oldest and longest-running British birth cohort study. We investigated how participation varied by lifetime and recent contact, health status, previous clinical feedback and study engagement, taking account of prior socioeconomic and cognitive characteristics. Overall participation and home visit participation remained high (94 and 80%, respectively) and there were no gender differences. Participation was higher in those with higher levels of prior contact and lower in those with the poorest health status. Having previously received clinical feedback on actionable blood results was associated with reduced home visit participation but other forms of clinical feedback were not associated with subsequent participation. Activities that fostered study engagement were associated with increased home visit participation. These findings inform strategies to maintain participation in life course studies.

**Electronic supplementary material:**

The online version of this article (doi:10.1007/s10654-016-0217-8) contains supplementary material, which is available to authorized users.

## Introduction

A life course approach to ageing investigates how biological, psychological and social factors from early life onwards, and across generations, affect health and its change with age in later life [1, 2]. There is growing evidence that biological ageing, manifesting as premature mortality, increased risk of chronic diseases, and decline in physical and cognitive capability with subsequent increases in functional limitations and difficulties with activities of daily living have their origins in environmental exposures and experiences earlier in life [3]. They also share certain underlying ageing processes post maturity that occur at the body system, cellular or molecular levels that lead to reduced physiological reserve [4]. There is also growing evidence that psychological and social wellbeing in later life have their origins earlier in life, albeit based on a somewhat different set of past exposures and experiences than those associated with biological ageing [5, 6]. Less well studied, from a life course perspective, are the lifetime determinants of the common health symptoms and conditions which are often the sequelae of biological ageing, such as chronic pain, incontinence and fatigue, which can impair quality of life and lead to a loss of independence.

Birth cohorts, and other life course studies, that follow population samples from early life into old age are valuable because they facilitate the study of lifelong health and its change across all phases of life, including periods of development and loss of capacity [7]. The MRC National Survey of Health and Development (NSHD), the oldest of the British birth cohort studies, whose participants reached age 70 in 2016, is a major resource for testing life course hypotheses on the extent of continuity and change in biological function and wellbeing, the development of disease risk and multi-morbidity, and their lifetime determinants and consequences [8]. The 24th and most recent follow-up, a postal questionnaire at 68 years followed by a home visit at 69 years, focused on morbidity and its consequences; and repeated functional and mental health assessments, updated reports of health and life circumstances, and collected a third blood sample. The postal questionnaire captured common health conditions, including chronic pain, fatigability, poor sleep quality and incontinence. The home visit captured a wide range of reported doctor diagnosed conditions; and collected more detail than before on functional limitations, difficulties with daily household and personal tasks, and use of health and social services (see Table S1, supplementary information).

Maintaining participation rates is important to reduce the risk of selective attrition biasing observed associations with lifetime risk factors [9–12], and because one of the aims of NSHD is to remain as representative as possible of the general population born in mainland Britain in the post war period [13]. Previous studies of older people suggest that permanent withdrawals and non-participation become increasing problems with advancing age [14–16]. We do not know if this also applies to lifelong participants in a birth cohort study. In NSHD and other studies, it has been shown that low educational attainment, mild cognitive impairment and socioeconomic disadvantage are key risk factors for drop out [13, 17–19]. Poor physical health can also increase risk of drop out, [16, 18, 19] although some studies with medical screening find the opposite [17].

In a birth cohort study with lifelong follow-up such as NSHD, some study members take part in all the sweeps whereas others decide not to participate in one or more sweeps without permanently withdrawing from the study. The level of prior contact is likely to be a good indicator of future participation, perhaps acting as a proxy for sense of belonging to a study, sense of duty, or because of other sociodemographic or behavioural attributes associated with participation. However few studies have looked at attrition over several waves in longitudinal studies; [14] those that have generally compare predictors of different types of attrition [17, 20].

Retention in longitudinal studies may be affected by the methods used by the study team to invite study members to take part in follow-ups, [21, 22] provide individual and general feedback, and encourage study belonging; and by the perceived quality of past interactions with the team. Since the 23rd follow-up, a clinical assessment at age 60–64, [8] the NSHD study team has purposefully had more interaction with study members than ever before, through letters reporting clinically relevant results or responding to individual comments or queries, by holding celebratory events for study members on their 65th and 70th birthdays, [23] and by involving some in media interviews and public events. We anticipated that this increased level of engagement would have beneficial effects on participation rates; although we were also concerned to examine whether increased feedback of clinically relevant findings, because of advancing age and the introduction of more detailed clinical assessments, was associated with reduced future participation, perhaps if those affected found this feedback worrying or disheartening.

In this paper, we report the participation patterns of NSHD study members at the 68–69 year follow-up and how these patterns relate to lifetime and recent contact, recent health status, receiving clinical feedback or being more engaged with the study at the preceding data collection. We test whether any observed associations were confounded by individual characteristics shown to affect participation at earlier ages [13, 24, 25].

## Methods

The NSHD is a representative sample of 5362 males and females who were born in England, Scotland and Wales in one week in March 1946 [8, 13, 24]. At the 24th follow-up, the target sample was 2816 study members still living in mainland Britain; this is the maximum sample used in the analyses. Of the remaining 2546 (47%) study members: 957 (18%) had already died, 620 (12%) had previously withdrawn permanently, 574 (11%) lived abroad, and 395 (7%) had remained untraceable for more than 5 years.

The postal questionnaire was sent out when study members were age 68; up to two reminder letters were sent to those who did not return a completed questionnaire. The home visit by a research nurse including blood sample collection took place at age 69 for the majority (97%), and at 70 years for the remainder. A short questionnaire, covering questions the research nurse asked at the home visit was posted to study members who did not wish to have a nurse visit. For this data collection, we obtained ethical approval from the NRES Queen Square REC (14/LO/1073) and Scotland A REC (14/SS/1009). This also included a protocol for collecting data on behalf of those who no longer had capacity to give consent (supplementary information).

### Prior contact

A measure of lifetime contact was derived by counting the number of data collections to the whole sample that each study member had taken part in, up to 60–64 years (maximum 23). A measure of the level of recent contact at the 60–64 year data collection distinguished: those who had completed a postal questionnaire and a clinic visit; those who completed a postal questionnaire and a home (rather than a clinic) visit; and a further three groups who had completed just one of the possible components (clinic or home visit or postal questionnaire). We also included two groups who had not taken part in the preceding data collection, either because they were unwilling or unable, or because they were only traced or had returned to live in mainland Britain after the 60–64 year data collection had finished.

### Recent health status

At age 60–64, we used a count of the number of clinical disorders (0, 1, 2, 3, 4, 5+) as a measure of recent health status [26]. To be included in this variable, study members required complete data on many health variables from the postal questionnaire and the home or clinic visit; therefore we included an item missing category. At age 68, general health was rated on the postal questionnaire using a five point Likert-scale from excellent to poor. Whether there was a longstanding illness and if this limited daily life was also reported. We used reported falls in the last year (categorised as none, 1, 2 or 3+) and four common health symptoms assessed using standardised scales: chronic pain, [27] fatigability, [28] incontinence, [29] and sleep quality [30, 31] (supplementary information). We also included item missing categories for these health measures which included between 10 and 337 participants.

### Previous clinical feedback

At 60–64 years, all clinical assessments (bloods, and cardiovascular and bone density imaging) were reviewed by the study doctor or relevant specialists, and participants were sent letters giving clinically relevant results. Participants with results outside normal ranges (but not actionable), were recommended to see the GP at their next appointment, those with actionable levels were advised to see the GP as soon as possible and the study doctor contacted the GP and participant directly if necessary [8]. Lifestyle advice was appended to bone density results as appropriate. We defined three variables indicating the feedback received on blood tests (actionable; outside normal range; all within normal range), cardiovascular imaging (letter indicating high level of plaque, or abnormal findings from echocardiogram), and bone density imaging (letter indicating osteoporosis, osteopenia or within normal range).

### Study engagement

We used three indicators to denote study engagement. First, during the data collection at age 60–64, study members in the target sample who gave extra comments or had queries received personal letters from the study director. Second, for those completing a clinic or home visit at age 60–64 we identified those who accepted our invitation to attend one of the four parties held to celebrate the NSHD’s 65th birthday or to provide memories of being in the study. A third variable denoted those in the latest target sample who responded positively when asked during the follow-up at age 68–69 if they would like to take part in public events or be interviewed by the media.

### Covariates: prior socioeconomic and cognitive characteristics

We chose socioeconomic and cognitive characteristics that were strongly associated with participation in this study at earlier sweeps [13]. These were: being in manual or non-manual occupations [taken at age 53 or earlier if missing (n = 34)]; educational qualifications (advanced level or equivalent, up to ordinary level or equivalent, none) by age 26; and a standardised measure of childhood cognitive ability measured at age 8, using tests of reading comprehension, pronunciation, vocabulary, and non-verbal reasoning [32]. Where data on childhood cognitive tests were missing at age 8 (n = 112), standardised scores from assessments at age 11 or 15 were used.

### Statistical analysis

**Fig. 1:**
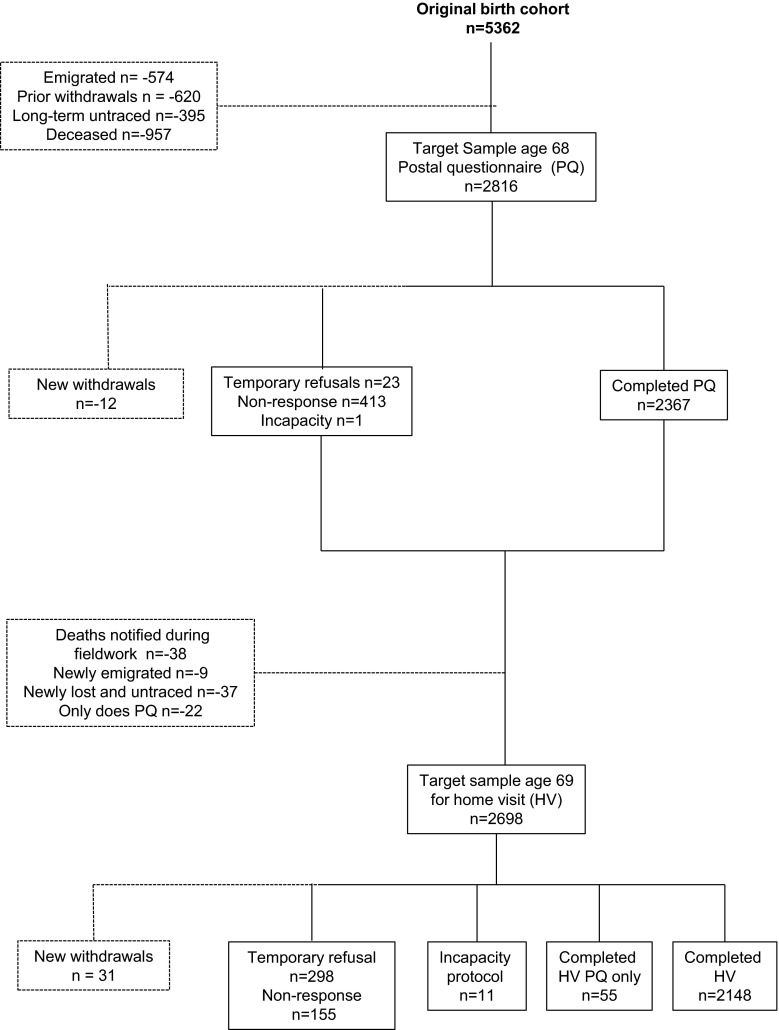
Deriving the target samples for the postal questionnaire at age 68 and the home visit at age 69 in the MRC National Survey of Health and Development

The *overall participation rate* for the 68–69 year data collection was calculated as those who completed a home visit or a home visit postal questionnaire, or a postal questionnaire, with the denominator being the original target sample. We calculated the 68 year *postal questionnaire participation rate as* the proportion completing the postal questionnaire out of the target sample. *The home visit participation rate* was the proportion with completed home visits out of the target sample for the home visit which excluded those who during the fieldwork period had died, emigrated, or become lost and remained untraced; and those who have previously requested not to be invited for a home visit but remain willing to complete postal questionnaires (Fig. 1).

How the overall participation rates at age 68–69 and the home visit participation rate at age 69 varied by prior contact, recent health status, clinical feedback, study engagement, and socioeconomic and cognitive characteristics was first assessed using Chi squared tests. Logistic regression models were then used to examine the associations of overall and home visit participation at 68–69 years with lifetime and recent contact, adjusting for sex and socioeconomic and cognitive characteristics. The logistic regression models were repeated for each indicator of health status, clinical feedback and study engagement that showed univariable associations with overall or home visit participation, adjusting for lifetime contact, sex and socioeconomic and cognitive characteristics. The clinical feedback and study engagement indicators were additionally adjusted for recent health status.

## Results

### Participation at 68–69 years

The postal questionnaire was completed by 2367 study members living in England, Scotland and Wales, representing 84% of the target sample for the postal questionnaire (Fig. 1). The home visit was completed by 2148 study members, representing 80% of the target sample for the home visit, and a further 2% (55) answered the interview questions on an additional postal questionnaire when a home visit was not possible. Of those invited to have home visit, 11 were unable to give informed consent and the incapacity protocol was initiated.

Overall, we obtained data from 2638 (94%) study members: 1978 completed a postal questionnaire and a home visit; 170 completed a home visit only; and 490 completed a postal questionnaires only. Of the 2148 who had a home visit, 96% were willing to have a blood sample taken, which was successfully obtained for 91%.

Participation rates did not vary by gender (Table 1). Being in a non-manual occupation, and having higher educational attainment and childhood cognitive ability were strongly associated with higher overall and home visit participation rates (Table S3, supplementary information).

**Table 1:** Overall and home visit participation at 68–69 years in the MRC National Survey of Health and Development by sex, prior participation and recent health status

Variable (maximum sample for overall/home visit participation)	Overall participation			Home visit participation		
	%	No. participants/maximum sample	*p* value	%	No. participants/maximum sample	*p* value
Sex (n = 2816/2698)			0.3			0.5
Male	90.3	1243/1376		80.0	1051/1312	
Female	91.5	1318/1440		79.1	1097/1386	
*Prior contact*						
No of lifetime contacts (n = 2816/2698)			<0.001			<0.001
23	96.5	871/903		86.2	760/882	
22	94.4	618/655		84.2	534/634	
21	88.4	335/379		72.5	261/360	
16–20	85.8	503/586		73.4	401/546	
1–15	79.9	234/293		69.6	192/276	
Contact at age 60–64 (n = 2816/2698)			<0.001			<0.001
PQ + CV	97.7	1486/1521		93.4	1397/1496	
PQ + HV	96.1	440/458		80.5	356/442	
CV only	88.0	110/125		84.0	100/119	
HV only	82.7	43/52		59.6	28/47	
PQ only	85.0	272/320		42.7	125/293	
Unwilling or unable (‘Temporary refusal’)	49.7	88/177		33.6	51/152	
Traced or returned to GB after 64 years	74.9	122/163		61.1	91/149	
*Recent health status*						
Number of clinical disorders at age 60–64 (n = 1979/1938)^a^			0.02			0.002
0	97.8	399/408		92.3	369/400	
1	98.2	391/398		94.1	369/392	
2	99.0	203/205		93.1	188/202	
3	95.2	120/126		87.9	109/124	
4	91.8	45/49		85.4	41/48	
5+	100.0	28/28		75.0	21/28	
Items missing^b^	96.7	740/765		88.2	656/744	
Self-rated health at age 68 (n = 2318)						<0.001
Excellent				88.2	195/221	
Very good				88.7	796/897	
Good				85.2	658/772	
Fair				78.4	268/342	
Poor				65.8	50/76	
Item missing^b^				80.0	8/10	
Limiting longstanding illness at age 68 (n = 2318)						0.031
Limiting longstanding illness				83.2	701/843	
Longstanding illness, not limiting				88.4	442/500	
No longstanding illness				85.6	812/949	
Items missing^b^				76.9	20/26	
Chronic pain at age 68 (n = 2318)^c^						0.253
None				84.8	852/1005	
Chronic widespread pain				83.9	208/248	
Chronic regional pain				87.7	604/689	
Other pain				84.1	280/333	
Items missing^b^				72.1	31/43	
Poor physical fatigability score at age 68 (n = 2318)^d^						0.672
Yes				86.9	761/876	
No				87.5	967/1105	
Items missing^b^				73.3	247/337	
Poor sleep quality at age 68 (n = 2318)^e^						0.397
Yes				85.9	619/721	
No				87.2	1103/1265	
Items missing^b^				76.2	253/332	
Incontinence at age 68 (n = 2318)^f^						0.053
More severe				86.4	248/287	
Mild				88.2	523/593	
None				84.1	1174/1396	
Items missing^b^				71.4	30/42	
Fallen in last year at age 68 (n = 2318)						0.101
3 + falls				82.7	86/104	
2 falls				89.3	109/122	
1 fall				89.4	236/264	
No falls				84.7	1521/1796	
Items missing^b^				71.9	23/32	

*PQ* postal questionnaire, *CV* clinic visit, *HV* home visit

^a^Maximum samples are participants with data from PQ and CV or HV at age 60–64 needed to distinguish those with up to 13 clinical disorders

^b^Those with items missing not included in calculation of *p* value

^c^Chronic pain assessed using criteria by American College of Rheumatology [27]

^d^Physical fatigability using the Pittsburgh Fatigability scale and a cut-point of 15 to denote high fatigability [28]

^e^Sleep quality using the Pittsburgh Sleep Quality Index using a cut point of 5 [30, 31]

^f^Incontinence using the ICIQ and a cut-point of 6 [29]. See also supplementary information

### Is participation at 68–69 years affected by prior contact?

Of those in the target sample at 68–69 years, a third (32%) had participated in all 23 previous data collections, over half (55%) in at least 22, and over two-thirds (69%) in at least 21 data collections.

Those with higher levels of lifetime contact or contact at the data collection at age 60–64 had higher overall and home visit participation rates at age 68–69 (Table 1). Those who had completed a postal questionnaire and a clinic visit at this preceding data collection had the highest overall participation and home visit participation rates at 68–69 years (98 and 93%); those choosing a home (rather than a clinic) visit, or not completing both the postal questionnaire and a visit, had lower subsequent participation. Those who were contacted but did not take part at the preceding data collection had the lowest subsequent participation rates (52 and 34%); and those who were traced or returned to live in Great Britain between these two data collections had participation rates of 75 and 61% respectively.

Lifetime and recent contact remained associated with overall and home visit participation at age 68–69 after mutual adjustment and adjustment for sex (Table 2, model 1). Higher lifetime contact and recent contact were associated with being in a non-manual occupational class, and having higher educational qualifications and childhood cognitive ability. Nevertheless, lifetime contact and recent contact remained independently associated with overall and home visit participation at 68–69 years after adjustment for these confounders (Table 2, model 2).

**Table 2:** Odds ratios (95% confidence intervals) of overall participation and home visit participation at 68–69 years by lifetime and recent contact

	Odds ratio (95% confidence intervals)			
	Overall participation (n = 2499)		Home visit participation (n = 2404)	
	Model 1	Model 2	Model 1	Model 2
Number of lifetime contacts (per category)	1.2 (1.0, 1.4)	1.2 (1.0, 1.4)	1.1 (1.0, 1.3)	1.1 (1.0, 1.3)
*p* value	0.02	0.02	0.01	0.01
Contact at age 60–64				
PQ + CV	1.0	1.0	1.0	1.0
PQ + HV	0.61 (0.32, 1.2)	0.83 (0.43, 1.6)	0.30 (0.21, 0.41)	0.37 (0.26, 0.52)
CV only	0.20 (0.10, 0.40)	0.22 (0.11, 0.44)	0.38 (0.22, 0.66)	0.41 (0.24, 0.72)
HV only	0.11 (0.05, 0.28)	0.16 (0.06, 0.40)	0.09 (0.05, 0.18)	0.12 (0.06, 0.23)
PQ only	0.13 (0.08, 0.21)	0.16 (0.10, 0.27)	0.05 (0.04, 0.07)	0.06 (0.04, 0.08)
Temporary refusal	0.03 (0.01, 0.05)	0.03 (0.02, 0.05)	0.04 (0.03, 0.06)	0.05 (0.03, 0.07)
Newly traced or returned to GB	0.09 (0.05, 0.16)	0.11 (0.06, 0.19)	0.14 (0.09, 0.22)	0.15 (0.10, 0.24)
*p* value	<0.001	<0.001	<0.001	<0.001

Model 1: Adjusted for sex, lifetime contact and recent contact (i.e. mutually adjusted)

Model 2: Model 1 + education qualifications, adult occupational class, childhood cognitive ability

### Is participation at 68–69 years affected by recent health status?

Having three or more clinical disorders at age 60–64 was associated with lower overall and home visit participation at age 68–69 but there was no difference in participation comparing those with fewer or no clinical disorders (Table 1). Those rating their general health as poor or fair at age 68 were less likely to have a home visit at age 69 (66 and 78% respectively) compared with those reporting that their health was good (85%), very good (89%) or excellent (88%). Those who reported a longstanding illness which limited activities were slightly less likely to have a home visit (83%) than those reporting no illness (86%) while those reporting a longstanding illness which was not limiting had the highest home visit participation (88%). Similarly, there was a tendency for those with milder symptoms or one or two falls to have higher participation than those with no incontinence, chronic pain or falls. Otherwise there were no associations, and no further analyses were conducted with these symptoms or falls.

Having three or more clinical disorders was not associated with overall participation in the sample with complete socioeconomic and cognitive data (Table 3, both models). However, having three or more clinical disorders and poor or fair self-rated health remained associated with lower home visit participation after adjustment for sex and lifetime contact (Table 3, model 1); these estimates were slightly weakened after mutual adjustment (model 2) and then after additionally adjusting for socioeconomic and cognitive characteristics (model 3). Longstanding illness was not associated with home visit participation after taking these factors into account.

**Table 3:** Odds ratios (95% confidence intervals) of overall and home visit participation at 68–69 years by recent health status

	Odds ratio (95% confidence intervals)				
	Overall participation (n = 1791)		Home visit participation (n = 1662)		
	Model 1	Model 2	Model 1	Model 2	Model 3
No. clinical disorders at 60–64 years					
0–2	1.00	1.00	1.00	1.00	1.00
3+	0.48 (0.18, 1.3)	0.54 (0.20, 1.4)	0.46 (0.26, 0.79)	0.56 (0.32, 0.98)	0.60 (0.34, 1.0)
*p* value	0.1	0.2	0.005	0.04	0.08
Items missing	0.45 (0.23, 0.88)	0.51 (0.26, 1.0)	0.52 (0.35, 0.76)	0.57 (0.39, 0.84)	0.65 (0.44, 0.97)
*p* value	0.02	0.06	0.001	0.004	0.03
Self-rated health at 68 years					
Excellent, very good, good			1.00	1.00	1.00
Fair or poor			0.40 (0.27, 0.59)	0.45 (0.30, 0.67)	0.53 (0.35, 0.79)
*p* value			<0.001	<0.001	0.002
Item missing			0.30 (0.03, 2.7)	0.32 (0.03, 2.9)	0.27 (0.03, 2.6)
*p* value			0.3	0.3	0.3

*p* value for each category versus the reference group

*Overall participation*

Model 1: Adjusted for sex and lifetime contact

Model 2: Model 1 + adult occupational class, educational qualifications and childhood cognitive ability

*Home visit participation*

Model 1: Each health variable separately adjusted for sex and lifetime contact

Model 2: Adjusted for sex, lifetime contact and both health measures (i.e. mutually adjusted)

Model 3: Model 2 + adult occupational class, educational qualifications and childhood cognitive ability

### Is participation at 68–69 years affected by previous clinical feedback?

Receiving a letter relating to actionable levels from the cardiovascular (8%) or bone density imaging (9%) was not associated with subsequent participation (Table 4). The majority of participants (81%) received a letter that noted at least one blood result outside the normal range, and one in ten (10%) received letters about actionable blood results; this last group had lower subsequent participation rates. The blood test that had the highest proportion of actionable results was the GGT liver function test which accounted for almost half (46%) of these results. The lower subsequent participation rates for those receiving actionable letters remained, albeit slightly attenuated, after adjusting for sex, and lifetime contact (Table 5, model 1), then for socioeconomic and cognitive characteristics (model 2), and finally for measures of recent health status (models 3a and 3b).

**Table 4:** Overall and home visit participation at 68–69 years by clinical feedback at 60–64 years and prior study engagement

Variable (maximum sample for overall/home visit participation)	Overall participation			Home visit participation		
	%	No. participants/maximum sample	*p* value	%	No. participants/maximum sample	*p* value
*Clinical feedback at age 60*–*64*						
Letter for blood test results (n = 2072/2022)^a^			0.009			0.02
Actionable blood results	92.9	210/226		84.6	181/214	
Results outside normal range	96.9	1636/1688		90.5	1497/1654	
All results within normal range	96.8	153/158		90.3	139/154	
Letter for plaque or abnormal echocardiogram (n = 1646/1615)^b^			0.6			0.7
Yes	97.7	126/129		93.6	116/124	
No	96.9	1470/1517		92.6	1381/1491	
Letter for bone density imaging (n = 1646/1615)^b^			0.2			0.6
Yes, osteoporosis threshold	96.7	148/153		94.6	140/148	
Yes, osteopenia threshold	97.8	717/733		92.8	669/721	
No	96.2	731/760		92.2	688/746	
*Prior study engagement*						
Letter to study members in response to queries at age 60–64 (n = 2731/2624)^c^			0.04			0.02
Yes	94.4	289/306		85.0	254/299	
No	90.9	2204/2425		79.4	1845/2325	
Attended 65th birthday party and/or provide memories about the study (n = 2156/2104)^d^			0.004			<0.001
Yes	98.6	477/484		96.9	462/477	
No	95.8	644/1672		87.2	1419/1627	
Willing to do take part in public events or speak to media (n = 2816/2698)			<0.001			<0.001
Yes	99.0	195/197		98.0	191/195	
No	90.3	2366/2619		78.2	1957/2503	

Denominators include those in the target sample at 68–69 years plus

^a^Blood results at age 60–64

^b^Clinic visit at age 60–64

^c^In the target sample at age 60–64

^d^Clinic or home visit at age 60–64

**Table 5:** Odds ratios (95% confidence intervals) of overall and home visit participation at 68–69 years by indicators of clinical feedback and study engagement

	Odds ratio (95% confidence intervals)							
	Overall participation				Home visit participation			
	No	Model 1	Model 2	Model 3a	No	Model 1	Model 2	Model 3b
Actionable blood test results at 60–64 years (yes vs. no)	1948	0.47 (0.25, 0.91)	0.49 (0.26, 0.95)	0.46 (0.23, 0.89)	1904	0.63 (0.41, 0.96)	0.65 (0.42, 1.0)	0.67 (0.42, 1.0)
*p* value		0.02	0.04	0.02		0.03	0.05	0.09
Letter to study member in response to queries at 60–64 years (yes vs. no)	2433	1.5 (0.88, 2.6)	1.3 (0.72, 2.2)	0.93 (0.51, 1.7)	2346	1.5 (1.1, 2.2)	1.3 (0.92, 1.9)	1.1 (0.71, 1.6)
*p* value		0.1	0.4	0.8		0.02	0.1	0.8
Attended 65th birthday party and/or provided memories (yes vs. no)	1948	2.7 (1.1, 6.3)	2.1 (0.88, 5.1)	1.9 (0.77, 4.5)	1904	4.1 (2.4, 7.0)	3.1 (1.8, 5.4)	2.8 (1.6, 4.9)
*p* value		0.02	0.09	0.2		<0.001	<0.001	<0.001
Willing to take part in public events or speak to media (yes vs. no)	2499	8.1 (2.0, 33.1)	5.2 (1.3, 21.3)	2.9 (0.68, 12.0)	2404	11.7 (4.3, 31.8)	8.4 (3.1, 22.8)	5.1 (1.9, 14.3)
*p* value		0.003	0.02	0.1		<0.001	<0.001	0.002

Model 1: Adjusted for sex and lifetime contact

Model 2: Model 1 + adult occupational class, educational qualifications and childhood cognitive ability

Model 3a: Model 2 + clinical disorders (categories: 0–2; 3+; item missing; questionnaire missing)

Model 3b: Model 3a additionally adjusting for self-rated health (categories: excellent, very good, good; poor or fair; item missing; questionnaire missing)

### Is participation at 68–69 years affected by previous study engagement?

Over one in ten (11%) received letters from the study director in response to their queries or comments at age 60–64; this group had higher participation rates at age 68–69 (Table 4). One in five (22%) took up the invitation to attend one of the four parties held to celebrate their 65th birthday or provided memories of being in the study, and 7% offered to take part in public events or be interviewed by the media. All these indicators of study engagement were associated with higher participation rates (Table 4). Being in a non-manual occupational class, and having higher educational qualifications and childhood cognitive ability were all associated with having queries or making comments requiring a special letter, or engaging in study social activities (*p* < 0.001). Having three or more clinical disorders at age 60–64 and fair or poor self-rated health at age 68 were associated with engaging less in study social activities (both *p* < 0.001), and with receiving a letter in response to a query (self-rated health only, *p* = 0.02).

Strong associations between taking part in 65th birthday activities or being willing to take part in public events or speak to the media and home visit participation remained, albeit attenuated, after adjusting for sex and lifetime contact (Table 5, model 1), then for socioeconomic and cognitive characteristics (model 2), and finally for recent health status (model 3b). The associations with overall participation were weaker. The associations between receiving a letter in response to a query and participation rates were slightly weaker in the sample with complete socioeconomic and cognitive data and were no longer associated with participation once all confounders were included in the model (Table 5, all models).

### Future participation and satisfaction

Of those completing a home visit, 98% expressed a preference to remain in the study should they lose capacity to give consent. Of those sent an evaluation form that was enclosed with their results letter, 72% were returned; 98% of these participants were very satisfied (89%) or satisfied (9%) with the visit overall.

## Discussion

### Maintaining participation

Participation in the 24th NSHD data collection at age 68–69 years was high; the postal questionnaire, nurse visit and overall participation rates (84, 80, 94%, respectively) for those living in Great Britain were even higher than the equivalent rates achieved at 60–64 years (81, 78, 84%). Ways we sought to achieve these rates were based on study members’ response at previous contacts. Being representative of the post war generation, study members appreciate annual birthday cards to maintain contact, the initial approach at each follow-up by formal letter, the style of any telephone or email contact, the personal approach we take to responding to queries, or to clinically relevant findings, and our efforts at accommodating their preferences in arranging visits, even where this has extended the fieldwork period. The team has experience in how many postal reminders or telephone calls achieve the best results, and the acceptable length of questionnaires and visits. With their consent, we feed forward information so that participants are not repeatedly asked certain questions (e.g. about parental death) if such information is already known.

Despite our concerns, there was no evidence that the pioneering clinic data collection at 60–64 years lowered future response for those attending the clinic—indeed evidence suggested the opposite. However, the increasingly biomedical nature of the data collections may deter some people from participating; we plan to ask if that was the case for those only completing postal questionnaires or not participating in the recent sweep (while remaining in the study). Our findings suggest these two groups need particular attention to encourage participation and avoid permanent withdrawal at a future sweep.

Despite the high overall and home visit participation rates, 25% of those who provided some information did not complete both the postal questionnaire and the home visit, and variables derived from several questions (such as some of the health status variables) also have item missing data. Missing data are problematic: one strategy for reducing missing data for participants who did not complete a postal questionnaire but did have a home visit was to feed forward essential questions not answered on the postal questionnaire onto the computer assisted nurse interview. In addition, where possible, nurses returned to complete clinical assessments that for reasons, such as faulty equipment, were not collected during the first visit (2%). The short questionnaire sent to those unable or unwilling to have a visit also captured missing information.

Missing data at previous sweeps is also a challenge for testing life course hypotheses. While NSHD has a high rate of continuous participation with over two-thirds of study members having missed no more than 2 out of 23 previous data sweeps, there is a group who come in and out of the study. Reasons for this include addresses not being kept up to date in previous years and study members living abroad for a period. The information presented indicates that careful tracing is worthwhile—between the two sweeps at age 60–64 and age 68–69, 159 study members returned and their overall and home visit participation rates were reasonably high. Recognising the presence of this group is important and requires careful consideration as many methods for dealing with losses to follow-up assume that losses are permanent.

At this latest follow-up there were no gender differences in overall participation rates; this is a change from previous NSHD follow-ups in adulthood where men have been less likely to participate [13, 24, 25]. This may be due to men becoming more willing to participate as they get older and possibly as a result of having more time after retirement. Alternatively, there may be more survival bias in men than women; 20% of men (n = 568) compared with 15% of women (n = 389) were not contacted for this latest data collection because they were already deceased.

In a long-term study, such as a birth cohort, higher levels of lifetime and recent contact are very informative about future participation, yet with few exceptions, [17] little attention is paid to prior contact in the literature on attrition in longitudinal studies. These variables mediated at least some of the socioeconomic and cognitive characteristics consistently shown to affect attrition.

### Recent health status, clinical feedback and participation

Recent health status was associated with participation rates, particularly the home visit. This becomes more of a problem as a birth cohort study matures into older ages and morbidity becomes widespread. At age 53, there was little evidence that health status was a key reason for low participation [13]. However the evidence from NSHD indicated that multi-morbidity by early old age and self-rated general ill-health had particularly adverse effects on participation. In contrast, there was no evidence that mild health symptoms affected participation—if anything the opposite. These findings suggest that distinguishing multi-morbidity or severe ill-health from less severe forms of ill-health may shed light on inconsistent associations between health status and attrition in longitudinal studies [17–19].

It was reassuring that feedback about clinically relevant results from cardiovascular and bone assessments that required further follow-up had no effect on future participation. While reporting of non-urgent blood results was not associated with subsequent participation, the reporting of actionable blood results was associated with reduced subsequent participation and this finding was only partially explained by socioeconomic, cognitive or health characteristics. This was not due to excess deaths as we only followed up those in the target sample at 68–69 years. Almost half of the actionable blood results were abnormal on a liver function test, which may have led to GPs recommending lifestyle or medication changes that were not appreciated. However, this is speculative as we were not able to collect information on how these results were followed up by the study members and their GPs.

### Study engagement and participation

In the last 6 years, it has been a major departure for the study team to provide opportunities for participants to meet each other and to invite them to share memories of the study and be involved in public and media events. We had to balance the risks of these activities having effects on life trajectories, or individuals regretting being identified as study members, against the benefits of feeling appreciated for their lifelong commitment. We had numerous requests from study members to celebrate key birthdays, and the birthday celebrations at age 65 were very well received, [23] as were the recent ones held to mark the 70th birthday. They also led to a much greater profile for the study (http://www.bbc.co.uk/news/uk-35699088). Our findings suggest that these types of activities are associated with higher subsequent participation even after allowing for the fact that those who take part, on average, have better socioeconomic circumstances, higher cognitive ability and better health. However, this evidence is not conclusive as there was no experimental design embedded in these engagement activities.

### Lessons learnt for maintaining participation into older age

We will use these findings to help the team to design the best ways to maintain future contact, and make subsequent home or clinic visits comfortable for those with multi-morbidity or those who generally prefer to complete postal questionnaires. We anticipate that there will be an important role for highly skilled research nurses attached to the study team to undertake the visits with participants who are vulnerable in terms of physical, mental or emotional capacity and who will accommodate individual needs of these study members (such as several short visits, or taking time to engage the participant and go at their pace). Increasing cognitive impairment will require more use of our protocols for those who no longer have the capacity to give consent, if their participation is to be retained. In NSHD, those lost many years ago are particularly hard to trace, so it is important to undertake tracing exercises at the earliest opportunity when a study member is first lost to follow-up.

In conclusion, evidence from this 70 year old birth cohort study shows that participation can remain high even when participants enter their seventh decade. This takes effort by the study team as participation rates appear to be favourably influenced by strategies that are tailored to appeal to their age, cohort, individual capacities and preferences. Activities that foster study engagement appear to be beneficial for subsequent participation.

## Electronic supplementary material

Below is the link to the electronic supplementary material.

Supplementary material 1 (DOCX 146 kb)
