# Supplementary material for: The MRC National Survey of Health and Development reaches age 70: maintaining participation at older ages in a birth cohort study
Source: Eur J Epidemiol. 2016 Dec 19;31(11):1135–47. doi: 10.1007/s10654-016-0217-8 (PMC5206260; doi:10.1007/s10654-016-0217-8)
Supplement: Supplementary file 1 — Supplementary material 1 (DOCX 146 kb) [file 10654_2016_217_MOESM1_ESM.docx]

**Medical Research Council (MRC) National Survey of Health and Development**

**Postal questionnaire and home visit 2014-15, age 68-69 years**

**Supplementary Information for:**

Kuh D, Wong A, Shah I, Moore A, Popham M, Curran P, Davis D, Sharma N, Richards M, Stafford M, Hardy R, Cooper R. The MRC National Survey of Health and Development reaches age 70: maintaining participation at older ages in a birth cohort study.

Corresponding author: Diana Kuh, MRC Unit for Lifelong Health and Ageing at UCL, [d.kuh@ucl.ac.uk](mailto:d.kuh@ucl.ac.uk)

Measures collected using the postal questionnaire and the home visit are shown in Table S1.

| Table S1. Measurements taken in NSHD at 68-69 years | |
| --- | --- |
| Mental status | Addenbrooke’s Cognitive Examination (ACEIII) |
| Tests of functional capacity, biological function, and anthropometric measures | Verbal memory, search speed and concentration, finger tapping, standing balance, grip strength, chair rises, walking test, spirometry, brachial blood pressure (Omron HEM-907)  Height, weight, waist and hip circumference. Weight changes in last year |
| Free-living physical activity | Tri-axial accelerometer worn at the waist for 7 days to measure vertical impact (GCDC X15-1c) |
| Non-fasting 25ml blood sample | Triglyceride, HDL cholesterol, total cholesterol, HbA1C, full blood count (including white cell count). DNA extraction, EDTA, citrate and serum aliquots stored at -80^o^C |
| Scales or questions from nurse home visit (*short questionnaire) | 19 doctor diagnosed diseases*, health symptoms*, hearing*, vision* and memory problems, pain and stiffness in hands and knees, knee injuries, functional limitations*, difficulties with basic and instrumental activities of daily living* (ADLsand IADLs), personal help with ADLs and IADLs* (current and future), prescribed regular medication*. AUDIT10, smoking*, GHQ28, parental death*, time spent caring for others*. Household income, total value of assets, perceived financial hardship |
| Scales or questions from postal questionnaire | Household size, housing tenure and car ownership, updated marital status, own/partner’s retirement, paid or voluntary work. CVD events and tests for chest pain/heart disease, hospital admissions, day surgery, outpatients, GP visits and health checks. Overall health and limitations, MRC questions on respiratory symptoms, , Rose angina, fracture history, pain (ACR criteria) and falls;incontinence (ICIQ), Pittsburgh fatigability scale, Pittsburgh sleep quality index, health behaviours (smoking, drinking, exercise and diet habits); Close Persons questionnaire,Pearlin Mastery Scale, Warwick Edinburgh Mental wellbeing scale, Personality (Big Five Inventory), religious and spiritual beliefs; spare time activities |

**Postal questionnaire at age 68**

The postal questionnaires were sent out in September 2014 with two reminders at approximately six-weekly intervals (Figure S1).

Figure S1. The cumulative percentage of postal questionnaires returned by date.

*
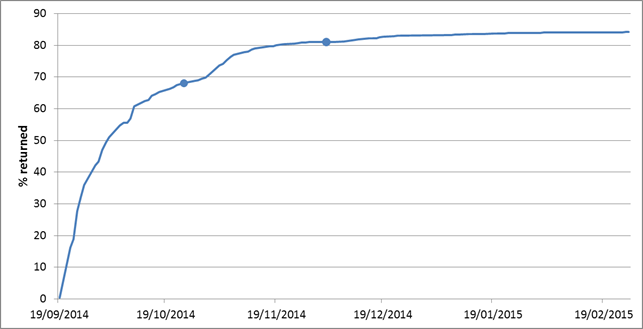

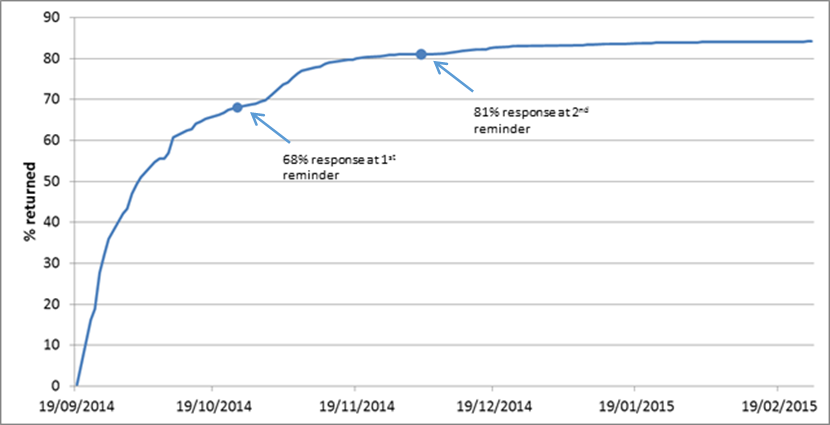
*

*Recent health status*

The clinical disorders at age 60-64 and their definitions included in this analysis can be found in Table 1 of the paper by Pierce et al in PLoS One[1]. We included the level 1 definition for cardiovascular disease, hypertension, raised cholesterol, renal impairment, diabetes, obesity, hypothyroidism, hyperthyroidism, anaemia, respiratory disease, liver disease, psychiatric problems and cancers. Below we provide further details about the derivation of the health symptoms at age 68 derived from data collected on the postal questionnaire and used in this analysis.

Chronic pain: Participants who reported any ache or pain which had lasted for one day or longer (excluding pain because of a feverish illness) were asked whether they had experienced this pain for at least 3 months, and then to shade the location using a four-view body manikin. Chronic widespread pain (CWP) was defined according to American College of Rheumatology criteria for fibromyalgia as pain present for 3 months or longer, above and below the waist; bilateral; and in the axial skeleton.[2] Participants who reported chronic pain but did not meet the CWP definition were classified as having chronic regional pain (CRP).

Fatigability: Fatigability, the level of physical and mental fatigue experienced when asked to perform a series of standardised tasks, was captured using the Pittsburgh Fatigability scale which asks the participant to assess on a scale of 0 to 5 the levels of physical and mental fatigue when undertaking ten activities of various intensities. We used the total physical fatigability scale (0-50) which has been validated against performance based measures of fatigability in the Baltimore Longitudinal Study of Aging,[3] and distinguished those with high physical fatigability (score of 15 or more) from others.

Incontinence: Incontinence was captured using the short form of the International Consultation on Incontinence Modular Questionnaire (ICIQ).[4] For this analysis, we distinguished those with a severity score of 1-5 and 6-21 to identify those with mild or more severe incontinence symptoms.

Sleep quality: Sleep quality was captured using the Pittsburgh Sleep Quality Index.[5,6]This has 19 items covering seven domains (sleep efficiency, sleep quality, sleep disturbance, use of sleep medication, daytime dysfunction, sleep latency and sleep duration). The index has a score from 0 to 21 and a cut-off of greater than 5 was used to define poor sleep quality.

**Home visit: main fieldwork processes**

The NSHD fieldwork team designed and implemented a three-day training programme for the nurses fromMDG and Kantar Public (formerly known as TNS-BMRB), the research partnership conducting the 2015 nurse home visit, and provided a 84-page manual detailing every step required for each protocol. All nurses were required to have satisfactory evidence of venepuncture skills.

The trained nurses were responsible for sending invitation letters from the study director, arranging appointments and assessingparticipants in their home. The NSHDfieldwork team supplied Kantar Public with invitation letters, a reply slip and an information brochure, providing details of the measures that would be taken and the feedback that participants and/or their GPs would receive. Non responders to the home invitation were telephoned by the Kantar Public CATI (Computer-Assisted Telephone Interviewing) team and followed up by NSHD as appropriate.

The nurses contacted the study member by telephone to arrange the appointment and to discuss any concerns (health, regular medication etc.). The nurses received home visit packs from the fieldwork team that contained paperwork and blood packs that were personalised for each study member prior to the appointment. Home visits lasted approximately 1.5 hours.

All electronic datawere returned to MDG by electronic transfer on a daily basis and subsequently transferred to the NSHD fieldwork team via secure FTP. Royal Mail was used to return all paperwork to the fieldwork team and blood samples to The Doctors Laboratory, London. A unique serial number wasassigned to each study member in the data collection. Only authorised NSHD staff can merge data linked to these numbers with the long-term unique serial number known to the study member.

*Home visit assessment*

The home visit was conducted by the research nurse using a CAPI (Computer-Assisted Personal Interviewing), the survey instrument was written as a computer program using bespoke software, and using a self-completion questionnaire. The CAPI showed the wording of questions, instructions to the nurse and included ‘soft’ checks, to remind the nurses of procedures or indicate improbable values, and ‘hard’ checks, which excluded values outside a pre-determined range or unacceptable combinations of response codes, facilitating data entry.

*Incapacity protocol*

Ethical approval from the NRES Queen Square REC (14/LO/1073) and Scotland A REC (14/SS/1009) included a new protocol for collecting data from those who no longer had capacity to give consent. This involves medical record review where prior consent for access had been given; an interview with a consultee who had previously been nominated by the study member to provide third-party into presentational features, and especially change in functionality; and where possible and where advance consent had been given, a brief assessment of the study member including mental status, affective symptoms, anthropometry, blood pressure and physical performance.Advanced consent was obtained during the data collection at age 60-64, by asking all participants to nominate and provide contact details for a consultee (typically a family member or close friend) in the event we are unable to contact them personally due to long-term physical or mental illness, or death. Over 90% of participants provided this consent. Advance consent was updated at the data collection at age 68-69.

*Biological samples*

Initial funding covered the costs of analytes required for the duty of care protocol. The other analytes were prioritised either on scientific grounds or on the viability of the analytes on storage; funding for these is being sought elsewhere by expert collaborators in conjunction with the research team. AW was responsible for the research protocols for collecting and storing the blood and urine samples needed to measure these analytes and for reporting clinically relevant results to participants and their GPs, in collaboration with NS (the study doctor).

Once informed consent had been given and a medical history taken, a 25ml blood sample was collected using the BD vacutainer system into 6 tubes. After allowing the serum sample to fully clot, the serum samples were spun in the home using a bench top centrifuge (Cole Parmer, UK)for at least 30 minutes at 3000rpm. All the blood samples were then posted by first class to The Doctors Laboratory, London,for processing according to a standardised protocol to generate 30 aliquots of serum and plasma for later analysis. A full blood count, HbA1c and lipid panel were undertaken at the analytical laboratory at The Doctors Laboratory on the day of receipt.The remaining frozen aliquots were couriered in batches to the ALSPAC laboratory in Bristol for future use.

In cases where action levels for these testswere met(Table S2),NSwas immediately informed and rang the study member and his/her GP if there was no known underlying medical condition explaining the abnormality. Action levels are those values at which any clinician would feel obliged to respond. These were largely based on the values used at MRC Human Nutrition Research (HNR) and Addenbrooke’s Hospital.

If any of the results were outside the normal range (but did not meet action levels) a decision was made in the context of all the clinical information about that study member, as to whether that person should be advised to make an appointment to see his/her GP at their next GP appointment. This was done to avoid unnecessary anxiety for the study member and work for the GP, and the decision was based on considerations such as the number and likely seriousness of any abnormalities, and whether the participant was under regular medical supervision. This advice accompanied the written reports containing the results for analytes commonly requested by GPs that were sent to participants at their request and to the GP where consent was given.

*Other assessments*

As the home visit assessments are not used in this paper, the study protocols for the physical and cognitive performance tests, spirometry, cardiovascular and mental status assessments, anthropometry, and assessment of vertical impact are included in relevant articles, published [7]or forthcoming.

**Table S2. Reference range and action levels for blood analytes at 68-69 years**

| **Measurement** | | | **Reference Range** | **Action level** |
| --- | --- | --- | --- | --- |
| Blood lipids (mmol/L) | | Total cholesterol | Optimal: <4.0  Moderately High: 4.0-6.0  High: >6.0 | >10 |
|  |  | HDL cholesterol | Normal: F ≥1.2, M ≥1.0  Low: F <1.2, M <1.0 |  |
|  |  | Total:HDL ratio | Normal: <4.5  High: >6.0 |  |
|  |  | Triglycerides | Normal: ≤2.2  High: >2.2 | >5 |
|  |  | LDL* | Normal: 0.0 - 3.0 |  |
| HbA1c (mmol/mol) | | | Normal: 20- 42 |  |
| HbA1c (%) | | | Normal: 0.0 - 6.0 | >10 |
| Blood counts | Haematocrit (%) | | Normal: 38 - 47 |  |
|  | Haemoglobin (g/L) | | Normal: M 130-180  Normal: F 115 - 165 | <100 or >200 |
|  | Platelet Count (10^9^/L) | | Normal: 150-450 | <50 or >1500 |
|  | Red Cell Count (10^12^/L)* | | Normal: F 3.95-5.51  Normal: M 4.4–5.8 |  |
|  | White Cell Count (10^9^/L) | | Normal: 4-11 | <2 or >30 |
|  | Neutrophils (10^9^/L) | | Normal: 2-7.5 | <0.5 or >20 |
|  | Lymphocytes (10^9^/L) | | Normal: 1.5-4.0 | <0.5 or >20 |
|  | Eosinophils (10^9^/L)* | | Normal: 0.0-0.4 |  |
|  | Basophils (10^9^/L)* | | Normal: 0-0.1 |  |
|  | Monocytes (10^9^/L)* | | Normal: 0.2-1.0 |  |

^M Male, F Female^

^* Results not reported back to study members^

Table S3. Overall and home visit participation at 68-69 years by socioeconomic and cognitive characteristics

|  | Overall participation | | | Home visit participation | | |
| --- | --- | --- | --- | --- | --- | --- |
|  | % | No. | p-value | % | n | p-value |
| Standardised childhood cognitive score |  |  | <.001 |  |  | <.001 |
| Top quintile | 93.8 | 519 |  | 83.4 | 500 |  |
| 2 | 94.6 | 519 |  | 87.6 | 498 |  |
| 3 | 93.3 | 519 |  | 81.4 | 499 |  |
| 4 | 89.4 | 519 |  | 75.9 | 493 |  |
| Bottom quintile | 85.9 | 519 |  | 70.9 | 499 |  |
| Educational qualifications by 26 years |  |  | <.001 |  |  | <.001 |
| A-level and above | 95.8 | 969 |  | 87.5 | 942 |  |
| Up to GCE | 90.7 | 750 |  | 80.5 | 717 |  |
| None | 87.2 | 936 |  | 71.3 | 889 |  |
| Adult social class |  |  | <.001 |  |  | <.001 |
| Non-manual | 94.0 | 1888 |  | 84.6 | 1821 |  |
| Manual | 86.1 | 894 |  | 70.6 | 849 |  |

**Supplementary references**

1. Pierce MB, Silverwood RJ, Nitsch D, et al. Clinical Disorders in a Post War British Cohort Reaching Retirement: Evidence from the first National Birth Cohort Study. PLoS ONE. 2012 Sep 19;7(9):e44857.

2. Wolfe F, Smythe HA, Yunus MB, et al. The American College of Rheumatology 1990 Criteria for the Classification of Fibromyalgia. Report of the Multicenter Criteria Committee. Arthritis Rheum. 1990 Feb;33(2):160-72.

3. Glynn NW, Santanasto AJ, Simonsick EM, et al. The Pittsburgh Fatigability scale for older adults: development and validation. J Am Geriatr Soc. 2015 Jan;63(1):130-5.

4. Avery K, Donovan J, Peters TJ, Shaw C, Gotoh M, Abrams P. ICIQ: a brief and robust measure for evaluating the symptoms and impact of urinary incontinence. Neurourol Urodyn. 2004;23(4):322-30.

5. Buysse DJ, Reynolds CF, III, Monk TH, Berman SR, Kupfer DJ. The Pittsburgh Sleep Quality Index: a new instrument for psychiatric practice and research. Psychiatry Res. 1989 May;28(2):193-213.

6. Buysse DJ, Reynolds CF, III, Monk TH, Hoch CC, Yeager AL, Kupfer DJ. Quantification of subjective sleep quality in healthy elderly men and women using the Pittsburgh Sleep Quality Index (PSQI). Sleep. 1991 Aug;14(4):331-8.

7. Hannam K, Deere KC, Hartley A, et al. A novel accelerometer-based method to describe day-to-day exposure to potentially osteogenic vertical impacts in older adults: findings from a multi-cohort study. Osteoporos Int. 2016 Oct 31.
